# Effect of blockade of the EGF system on wound healing in patients vaccinated with CIMAvax® EGF

**DOI:** 10.1186/1477-7819-11-275

**Published:** 2013-10-15

**Authors:** Aymara Fernández Lorente, Soraida Acosta Brooks, Elia Neninger Vinageras, María del Carmen Barroso Alvarez, Barbara Wilkinson Brito, Mayelin Troche Concepción, Liana B Martínez Pérez, Carmen E Viada González, Tatiana Crespo Diaz, Angel Raymundo Casacó Parada

**Affiliations:** 1 Center of Molecular Immunology, Clinical Research Direction, PO Box: 16040, Havana 11600, Cuba; 2 Saturnino Lora Hospital, Santiago de Cuba, Cuba; 3 Hermanos Ameijeiras Hospital, Havana, Cuba; 4 Benéfico Jurídico Hospital, Havana, Cuba

**Keywords:** CIMAvax® EGF, Wound healing, EGF, EGFR

## Abstract

**Background:**

The epidermal growth factor receptor (EGFR) signaling system is frequently unbalanced in human malignancies due to increased ligand production, receptor overexpression, receptor mutations, and/or cross-talk with other receptor systems. For this reason, the EGFR is an attractive target for anticancer therapy. The epidermal growth factor also plays an important role in regulating multiple facets of cutaneous wound healing, including inflammation, wound contraction, proliferation, migration, and angiogenesis. In the Center of Molecular Immunology, a cancer vaccine is produced (CIMAvax® EGF) that blocks the binding of EGF to its receptor. This blockade causes a significant inverse association between the anti-EGF antibody titers and EGF concentration. Around 1,500 patients with non-small cell lung cancer have been treated, showing that this vaccine is safe, immunogenic, increases survival and improves quality of life. Taking into account the therapeutic benefits of CIMAvax® EGF vaccination and the role of EGF-EGFR system in the wound healing process, we decided to conduct a retrospective research with the aim of determining the effect to the CIMAvax® EGF vaccine on the wound healing process in patients undergoing surgical treatment.

**Methods:**

Medical records of 452 vaccinated patients were reviewed and only six patients receiving surgical treatment were identified. Further information about these six patients was obtained from source documents, including medical records and operative reports using an observational list that included different variables. Post-surgical wound healing complications were identified using the National Cancer Institute Common Toxicity Criteria for Adverse Events (NCI-CTC) version 3.0.

**Results:**

None of the six patients operated on presented adverse events related to the wound healing, that is to say, no wound dehiscence, wound infection, delayed wound healing, fistula formation, abscess formation or hemorrhage/bleeding associated with surgery during treatment with CIMAvax® EGF occurred.

**Conclusions:**

These results suggest that the use of CIMAvax® EGF does not produce a deleterious effect in the wound healing process.

## Background

The epidermal growth factor receptor (EGFR) signaling system is frequently unbalanced in human malignancies due to increased ligand production, receptor overexpression, receptor mutations, and/or cross-talk with other receptor systems [1-3]. Changes in EGFR status have been linked to the development and maintenance of a malignant phenotype and correlated with poor clinical prognosis [4]. For this reason, the EGFR is an attractive target for anticancer therapy [5].

On the other hand, a series of experimental and clinical studies have demonstrated a positive effect of EGF on wound repair, regulating multiple facets of cutaneous wound healing, including inflammation, wound contraction, proliferation, migration, and angiogenesis [6-9].

CIMAvax-EGF is a therapeutic cancer vaccine produced in the Center of Molecular Immunology, composed by human recombinant epidermal growth factor (EGF) conjugated to the carrier protein P64K from Neisseria meningitides and emulsified in Montanide ISA 51 (Seppic.http://www.seppic.com/index.html Puteaux Cedex, FRANCE). The vaccine induces antibodies against EGF, one of the most important ligands of the EGFR that would block EGF-EGFR binding. This blockade causes a significant inverse association between the anti-EGF antibody titers and EGF concentration [10]. So far, six clinical trials have been concluded and around 1,500 patients have been treated, showing that this vaccine is safe, immunogenic, increases survival and improves quality of life [10-14]. It has also been shown that chronic use of CIMAvax® EGF ensures a better response without evidence of accumulative toxicity [15]. However, the chronic use of this vaccine increases the possibility that patients will need surgical procedures during the vaccine treatment period.

Taking into account the therapeutic benefits of CIMAvax® EGF vaccination and the role of the EGF-EGFR system in the wound healing process, we decided to conduct a retrospective research with the aim of determining the effect of the CIMAvax® EGF vaccine on the wound healing process in patients undergoing surgical treatment.

## Methods

### The EGF cancer vaccine

The CIMAvax® EGF vaccine is produced in the Center of Molecular Immunology, Havana, Cuba, in manufacturing facilities validated according to the European Medicines Agency (EMA) Good Manufacturing Practice standards. It is compound or composed by human recombinant EGF produced in yeast, and chemically conjugated to the P64K Neisseria meningitidis recombinant protein, produced in E. coli [15,16]. For protein conjugation, glutaraldehyde (0. 05%) is added to the protein mixture allowing the reaction for one hour; then, the conjugated moiety is purified by ultra-filtration/diafiltration and sterile filtered [17]. The vaccine is composed of the chemically conjugated EGF-P64k containing two EGF molecules per P64k molecule. For preparations in which alum was used as adjuvant, conjugates were mixed after filtration with 2 mg/dose of alum. Adsorption is achieved by constant stirring at room temperature for one hour under sterile conditions. Montanide ISA 51 (Seppic.http://www.seppic.com/index.html Puteaux Cedex, FRANCE) is used as the adjuvant. The conjugate is mixed with an equal volume of the adjuvant until emulsification immediately before injection. Each vaccine dose contains 1 mg of protein. The product is released in the Quality Control Direction of the Center of Molecular Immunology according to manufacturer specifications which include among others, HPLC for molecular species, potency measured as immunogenicity in vaccinated mice (in vivo test), sterility and appearance [18].

This EGF-based cancer vaccine obtained registration in 2008 by Sanitary Registration from The Cuban Regulatory Agency, CECMED, for advanced non-small cell lung cancer (NSCLC) at stage IIIB-IV (surgical treatment nontax). This was the first registration of a therapeutic vaccine in Cuba and also the first registration of a lung cancer vaccine in the world [14]. It also was registered in Peru [19].

### Patient selection and methods

The cohort of patients included in this analysis comes from nine different clinical trials; all have been diagnosed with advanced NSCLC patients (stage IIIB-IV). The results of five of these clinical trials have been previously published [10-14,18,20-22]. These studies were approved by the Institutional Review Boards of the participating centers and authorized by the Cuban Regulatory Agency. All patients provided informed consent before inclusion in the clinical trial.

To identify patients who had undergone surgical treatment, medical records of 452 patients were reviewed. After identification of patients (ID) who had received surgical treatment, further information was obtained from source documents, including medical records and operative reports using an observational list that included different variables, such as: ID of patients, age, race, toxic habits, personal history, medication use, time between receiving vaccine and surgery, time between last dose of vaccine and surgery, type of surgery, time between surgery and next vaccination, treatment schedule and adverse events related to wound healing. The review of the medical records included patient follow-up period as part of the clinical trials.

Although we took into account some of the risk factors for reduced proper healing: age and race; harmful habits such as smoking habit, alcoholism and drug abuse; personal history (obesity, paraplegia, coagulopathies, coronary artery disease, peripheral vascular disease, immunosuppression, malnutrition and diabetes mellitus); use of certain drugs (anticoagulants, antiplatelet drugs, steroids, NSAIDs, chemotherapeutic drugs and radiation therapy) [23,24], the presence of these risk factors was not considered to constitute exclusion criteria for this study.

Surgery was classified as minor surgery and major surgery [25]. Minor surgery included any surgical procedure not involving general anesthesia or respiratory assistance. Major surgery included any surgical procedure involving anesthesia or respiratory assistance; all operations involving opening body cavities or in which severe hemorrhage was possible; all potentially life-threatening conditions; and any procedure with the potential to induce permanent anatomic (physical) or physiological impairment and/or associated with orthopedics or extensive tissue dissection.

### Assessment of postsurgical wound healing complications

Postsurgical Wound Healing Complications were identified using the National Cancer Institute Common Toxicity Criteria for Adverse Events (NCI-CTC) version 3.0. Search terms included, wound dehiscence, wound infection, delayed wound healing, fistula formation, abscess formation and post-operative hemorrhage/bleeding (post-operative period is defined as ≤ 72 hours after surgery).

## Results

From the 452 clinical records reviewed in the nine clinical trials, only six patients out of three clinical trials underwent surgical treatment. One of them underwent two major surgical procedures. None had wound healing complications.

A detailed description of each individual patient who underwent a surgical process is presented below and Table 1 summarizes these results.

**Table 1:** Description of the behavior of different studied variables

**Patient**	**Age**	**Race**	**Toxic habits**	**Personal history**	**Concomitant medications that could interfere with proper healing**	**Time receiving vaccine until surgery**	**Time between last vaccination and surgery**	**Type of surgery**	**Time between surgery and next vaccination**	**Post-surgical wound healing complications (wound dehiscence, wound infection, delayed wound healing, fistula formation, abscess formation and post-operative hemorrhage/bleeding)**
1	63	White	Ex-smoker (30 cigarettes per day for 30 years)	Moderate chronic obstructive pulmonary disease	No	35 months	22 Days	Right cordectomy (Major surgery)	13 Days	No
2	74	White	Ex-smoker (20 cigarettes per day for 55 years)	No	No	45 months	27 Days	Total laryngectomy (Major surgery)	93 Days	No
3	59	Afro-American	Ex-smoker	No	No	8 months	32 Days	Thoracotomy (Major surgery)	NA	No
							50 Days	Lobectomy (Major surgery)		
4	61	White	Ex-smoker	No	No	7 months	51 Days EGF	Craniotomy (Major surgery)	NA	No
							57 Days hR3			
5	66	White	Ex-smoker (44 years)	Chronic gastritis; Chronic bullous emphysema	No	32 months	11 Days	Inguinal hernia surgery (Major surgery)	17 Days	No
6	75	Afro-American	Ex-smoker	No	No	5 months	1 Day	Minimum pleurotomy (Minor surgery)	31 Days	No

NA (not applicable): no possible resumption of CIMAvax® EGF treatment.

hR3 (nimotuzumab): an anti-EGFR monoclonal antibody.

### Patient 1

A 63 year-old man, with moderate chronic obstructive pulmonary disease. He was diagnosed with squamous cell carcinoma of the right lung in May 2003 and started vaccination in the same month. Three years later he was diagnosed with a right vocal cord polyp. A biopsy of the lesion showed squamous cell carcinoma and he underwent a right cordectomy (major surgery). Twenty-two days elapsed between last vaccination and surgery. Thirteen days after surgery the patient resumed vaccination without wound healing complications. The patient received the following treatment schedule as part of the clinical trials: cyclophosphamide (200 mg/m^2^ body surface (SC)) 72 hours before the first vaccination, two doses (every 14 days) of CIMAvax® EGF: 2,4 mg (0.6 mg per each injection site) given intramuscularly. Eighteen days after the second vaccination dose: chemotherapy: cisplatin 100 mg/Kg vinblastine 6 mg/Kg (every 21 days for four to six cycles) and one month after CIMAvax® EGF: 2,4 mg (0,6 mg per each injection site) given intramuscular monthly as re-immunizations doses.

### Patient 2

A 70-year old man diagnosed with squamous cell carcinoma of the left lung in July 2003. He started vaccination in the same month and 45 months after initiation of therapy, he underwent a total laryngectomy (major surgery) for a left vocal cord tumor extending to the subglottis (squamous cell carcinoma). He continued using CIMAvax® EGF until 27 days before surgery and resumed this treatment 93 days after the surgery. His skin wound healing was unremarkable. This patient had received the same treatment schedule as patient 1.

### Patient 3

A 58-year old man required thoracotomy (major surgery) without surgical resection of the lung nine months after starting CIMAvax® EGF for adenocarcinoma of the lung. Eighteen days after first surgery he underwent lobectomy (major surgery). The time between last vaccination and surgery was 32 days and 50 days respectively. Re-vaccination with CIMAvax® EGF was not possible due to deterioration of performance status. The wounds healed without any complications.

This patient received cyclophosphamide (200 mg/m^2^ SC) 72 hours before the first vaccination, CIMAvax® EGF: 2.4 mg (0,6 mg per each injection site) given intramuscularly every 14 days as induction doses and then monthly as re-immunizations doses.

### Patient 4

A 61 year-old man diagnosed with NSCLC was treated with CIMAvax® EGF plus nimotuzumab (an anti-EGFR monoclonal antibody) during seven months. Vaccination was interrupted after performance status deterioration. Fifty-one days after the last dose of CIMAvax® EGF and fifty-seven days after the last dose of nimotuzumab, he underwent a craniotomy (major surgery) due to a brain metastasis without delayed wound healing.

The patient was treated with cyclophosphamide (200 mg/m^2^ SC) 72 hours before the first vaccination, CIMAvax® EGF: 2.4 mg (0,6 mg per each injection site) given intramuscularly every 14 days for three months and then monthly as re-immunization doses plus nimotuzumab: 200 mg intravenous as 12 weekly induction doses and then every 14 days.

### Patient 5

A 66 year-old man with previous history of chronic gastritis and chronic bullous emphysema. He underwent inguinal hernia surgery (major surgery) thirty-two months after starting CIMAvax® EGF treatment for NSCLC. He was on treatment with CIMAvax® EGF without any interruption (11 days elapsed between last vaccination and surgery and 17 days after the patient received the next administration). The wound healed without any complications.

As part of the clinical trials this patient received cyclophosphamide (200 mg/m^2^ SC) 72 hours before the first vaccination, CIMAvax® EGF: 2.4 mg (0.6 mg per each injection site) given intramuscularly every 14 days for three months and then as monthly re-immunizations doses.

### Patient 6

A 75 year-old woman required a minimum pleurotomy (minor surgery) as a consequence of a pleural effusion five months after starting CIMAvax® EGF for NSCLC. She continued to use CIMAvax® EGF until the day before surgery and resumed this vaccination 31 days after the surgery. Her skin wound healing was unremarkable. This patient received the same treatment schedule as that of patient 5 .

## Discussion

Growth factors represent the intercellular signaling that orchestrates the complex sequence of cell migration, division, differentiation, and protein expression during wound healing and they are expressed in varying levels by the cells involved with healing. As an essential part of skin regeneration when injury has taken place, EGF works by attracting cells to a wound site in order to begin the repair process. The vital protein is released by platelets during the inflammation stage of healing. This protein supports cell renewal by assisting in the synthesis of proteins, increasing circulation, mitosis, the number of fibroblasts, the accumulation of collagen and blood-vessel formation [26].

Several clinical trials have been carried out with CIMAvax® EGF vaccine. In more than 1,500 patients treated, there are no reports of wound dehiscence, wound infection, delayed wound healing, fistula formation, post-operative hemorrhage or any other adverse events associated with post-surgical wound healing complications [10-12], [20-22], [27], but no analysis or in-depth discussion have been previously reported.

A previous non-clinical study investigated the possible role of the EGF-vaccine in the croton-oil-induced ear edema and, in the wound healing experimental animal models, concluded that the EGF vaccination in mice decreased the normal croton-oil-induced inflammation response without apparent impairment in tissue healing [28].

The use of anti-cancer target agents is increasing, such as the anti-cancer target drugs blocking the EGF/EGFR system. It is common to utilize a multi-modal treatment approach towards solid tumors, often including surgical resection, and it has become apparent that some targeting agents can impair wound healing or cause increased risk of perioperative complications, however, a recent article reviews many of the targeting agents used in solid tumor, suggesting that up to now, evidence of targeting agents causing surgical complications is limited [29].

Casacó et al. [30] reported an extensive review about the effects of anti-epidermal growth factor/epidermal growth factor receptor therapeutic anti-cancer drugs on the wound healing process. This review included the monoclonal antibodies cetuximab, panitumumab and nimotuzumab; the small tyrosine kinase molecules erlotinib and gefitinib; the EGF-based cancer vaccine CIMAvax®; and the EGFR-based cancer vaccine HER-1 vaccine, concluding that apparently, there are no deleterious effects of the anti-EGF/EGFR drugs in the wound healing post-operative process.

We are reporting the first clinical study of the wound healing risk associated with surgery after the use of the therapeutic cancer vaccine, CIMAvax® EGF. The results of this study show that the following variables: the time using the vaccine before surgery (a mean time of 22 months), the time between the last vaccination and surgery (the mean time was 36 days) and the time between surgery and the next vaccination (a mean time of 38 days) did not interfere with proper wound healing. Five of the six patients operated on started the vaccination six months before surgery and three patients were vaccinated without discontinuation treatment for more than two years before surgery. One of them (patient 1) was vaccinated the day before surgical treatment. The majority of patients (four of them) resumed vaccination in a period less than one month after surgery (See Table 1).

Other endogenous growth factors different from EGF, such as platelet-derived growth factor (PDGF), transforming growth factor-alpha (TGF-α), transforming growth factor-beta (TGF-β), basic fibroblast growth factor (bFGF), heparin-binding EGF (HB-EGF), and amphiregulin (AR) also have substantial impact on both epidermal and dermal wound repair [31-33]. Special interest is focused on TGF-α, HB-EGF and AR, which also operate by interacting with the EGFR, and could be responsible for the normal final wound healing process in these patients.

García et al. [13] measured the antibody titers against TGFα (an important growth factor involved in the healing process) in serum of patients treated with CIMAvax® EGF, looking for the possibility of cross-reactivity induced by EGF vaccination and they concluded that no induction of anti-TGFα antibodies and no reduction of TGFα concentration occurred as a consequence of EGF vaccination.

Previous studies have shown that serum EGF concentrations decreased to undetectable levels in all patients vaccinated with CIMAvax® EGF [22]. This vaccine causes a significant inverse correlation between antibody titers and serum EGF concentrations, indicating that vaccination with EGF results in a deprivation of circulating EGF [12,13]. Additionally, the patients maintain high anti-EGF antibody titers throughout the time the vaccination is active, without evidence of immune response exhaustion [15] and the patients who underwent surgical treatment did not have any wound healing complications.

## Conclusion

The results of this study suggest that apparently the use of CIMAvax® EGF does not produce a deleterious effect in the wound healing process as evidenced by the fact that none of the patients presented adverse events related with the wound healing process; however we emphasize, considering the small number of patients who underwent surgical treatment, that patients receiving CIMAvax® EGF treatment who undergo surgery should be carefully monitored during the post-surgical period.

## Abbreviations

AR: Amphiregulin; bFGF: Basic fibroblast growth factor; CECMED: Centro para el Control Estatal de Medicamentos, Equipos y Dispositivos Médicos; EGFR: Epidermal growth factor receptor; EGF: Epidermal growth factor; EMA: European Medicines Agency; HB-EGF: Heparin-binding EGF; HPLC: High-performance liquid chromatography; ID: Identification; NSAID: Non-steroidal antiinflammatory drug; NSCLC: Non-small cell lung cancer; SC: Body surface; NA: Not applicable; PDGF: Platelet-derived growth factor; TGF-α: Transforming growth factor-alpha; TGF-β: Transforming growth factor-beta.

## Competing interests

The authors declare that they have no competing interests

## Authors’ contributions

SAB, ENV, MCBA and TCD participated in acquisition of data and analysis and interpretation of results. BWB, MTC, LBMP and CEG have made contributions to acquisition of data and have been involved in drafting the manuscript. ACP has made substantial contributions to the conception and design of this study and has been involved in revising it critically for important intellectual content. All authors read and approved the final manuscript.
